# Neighbourhood crime and smoking: the role of objective and perceived crime measures

**DOI:** 10.1186/1471-2458-11-930

**Published:** 2011-12-14

**Authors:** Martine Shareck, Anne Ellaway

**Affiliations:** 1 Département de médecine sociale et préventive, Université de Montréal, Montreal, Canada; 2 Institut de recherche en santé publique de l'Université de Montréal, Montreal, Canada; 3 Centre de recherche du centre hospitalier de l'Université de Montréal, Hôtel-Dieu, Montreal, Canada; 4 Medical Research Council Social & Public Health Sciences Unit, Glasgow, UK

**Keywords:** Smoking, Tobacco, Neighbourhood, Perceived crime, Police-recorded crime, Contextual effects

## Abstract

**Background:**

Smoking is a major public health problem worldwide. Research has shown that neighbourhood of residence is independently associated with the likelihood of individuals' smoking. However, a fine comprehension of which neighbourhood characteristics are involved and how remains limited. In this study we examine the relative contribution of objective (police-recorded) and subjective (resident-perceived) measures of neighbourhood crime on residents' smoking behaviours.

**Methods:**

Data from 2,418 men and women participating in the 2007/8 sweep of the West of Scotland Twenty-07 Study were analyzed. Smoking status and perceived crime were collected through face-to-face interviews with participants. Police-recorded crime rates were obtained from the Scottish Neighbourhood Statistics website at the datazone scale. Adjusted odds ratios and 95% confidence intervals were estimated for the likelihood of current smoking using logistic regression models. Adjusted mean daily amount smoked and F statistics were calculated using general linear models. Analyses were conducted for all respondents and stratified by sex and age cohort.

**Results:**

Compared to individuals living in low crime areas, those residing in an area characterized by high police-recorded crime rates or those perceiving high crime in their neighbourhood were more likely to be current smokers, after controlling for individual characteristics. The association with smoking was somewhat stronger for police-recorded crime than for perceived crime. Associations were only slightly attenuated when adjusting for either the objective or subjective crime measures, suggesting that these indicators may exert an independent influence on the risk of smoking. Stronger effects were observed for women compared to men. Police-recorded crime rates were more strongly related to smoking status among older respondents than among the younger cohort, whereas the strongest effect for perceived crime was observed among younger participants.

**Conclusions:**

Our findings highlight the relevance of paying attention to both objective and perceived neighbourhood crime measures when aiming to prevent smoking.

## Background

Smoking is a major public health challenge and the principal risk factor for cancer, cardiovascular diseases and tuberculosis. Together, these are responsible for 70% of all deaths that occur in adults over 30 years-old worldwide[1]. As such, smoking is the main modifiable cause of premature death [1] and exposures that influence smoking and could be targeted in preventive efforts should be identified.

It is increasingly recognized that where people live influences their health behaviours, including smoking [2-11] and there have been calls for a better understanding of the mechanisms involved in producing these "place effects" [10]. A small number of studies have found aspects of the local neighbourhood such as safety and crime to be related to smoking [8,11-17] even after controlling for residents' individual characteristics. Crime and safety measures represent socio-cultural features of neighbourhoods which may act as chronic stressors [18] and as such, may influence smoking through pathways such as stress or psychological well being.[10,19] Most studies of crime-related exposures and smoking have investigated subjective measures of safety as perceived by respondents [8,12-15], or municipal authorities [17] while few have relied on more objective measures of crime such as rates recorded by police authorities [11,16]. We borrow from Weden *et al*. (2008) the terms *subjective *measure which refers to an "individual-level assessment of a resident's neighbourhood" and *objective *measures which refer to "area-level indicators that can be characterized independent of a resident's perception" of his neighbourhood [[19] p.1257]. Since objective and subjective crime indicators may be tapping into different concepts [19,20], it appears relevant to study both exposures in the same study. We found no study on smoking that had done so and thus sought to fill this gap found in the literature by studying how both objective (police-recorded) and subjective (resident-perceived) measures of crime influenced smoking, as well as whether, and if so how, each altered the other's association with the likelihood of smoking and with smoking intensity.

The study's objective was to investigate the association between smoking behaviour and neighbourhood crime measured in two ways: as recorded by police and as perceived by participants. Associations were investigated separately for men and women since some studies have found gender differences in the magnitude of associations between individual health and health behaviours and experience or perception of neighbourhood conditions [5,8,21-24]. Analyses stratified by age group were also conducted since neighbourhood exposures may be perceived differently by younger versus older individuals and exert a differential effect on their health [23,25]. For example, younger working-age cohorts may be more mobile than older groups. As such they may be less exposed to, and influenced by, their residential environment than less mobile groups [26,27].

## Methods

### Study population

The Twenty-07 Study has been following people in three age cohorts (born around 1932, 1952 and 1972) sampled from the Central Clydeside Conurbation, West of Scotland, for 20 years [28]. In this paper, data from the fifth sweep of the study conducted in 2007/8 were used (n = 2,604 respondents, of whom 2,459 resided in the Greater Glasgow area at the time of interview and were considered for the present analyses). Respondents who participated at baseline have been shown to be representative of the general population of the sampled area [29]. Ethics approval was gained for each wave from the NHS and/or University of Glasgow Ethics Committees. All analyses were conducted in the summer 2010.

### Individual-level variables

Individual-level measures were collected through face-to-face interviews conducted in participants' home by trained nurse interviewers. Information was collected on participants' socio-demographic characteristics (education, income, occupation, marital status), health conditions and behaviours including smoking, alcohol intake and physical activity, and everyday life including respondents' perceptions of neighbourhood conditions. Age was operationalized as a categorical variable corresponding to the study cohort (aged around 35, 55 and 75 when interviewed in 2007/8). Socio-economic status (SES) was operationalized with the head of household's social class defined using the 1980 Registrar General's classification: I (professional), II (intermediate), IIINM (skilled non-manual), IIIM (skilled manual), IV (partly skilled manual) and V (unskilled manual) [30]. Employment status indicated whether the participant was in employment or not at the time of interview. The unemployed included the retired, women on maternity leave and participants temporarily or permanently sick. The final sample for analysis is composed of 2,418 individuals (1,073 men and 1,345 women) for whom complete data for the variables of interest were available.

Perceived crime was used as an individual, resident-based subjective indicator of neighbourhood crime. It was measured by summing, for each participant, responses to the question: "Around where you live would you say that any of the following problems exist?" and included the items vandalism, burglaries, assaults and disturbances by children and youth. Problems were measured on a three-point scale using "not a problem" (score 1), "a minor problem" (score 2) and "a major problem" (score 3). A score was constructed by summing responses to each of the four items and scores were subsequently divided into quartiles, following their natural distribution in the sample. Higher scores indicated higher perceived crime.

### Neighbourhood-level variables

Neighbourhoods were operationalized as datazones, the key small area statistical geography in Scotland. Scotland is divided into 6,505 datazones comprising on average 500 to 1,000 inhabitants. Datazones have been created using 2001 Census data so as to be relatively socially homogeneous. In general, they also respect physical boundaries and natural communities [31]. Participants in our sample were distributed across 1,256 datazones with an average of two individuals per zone (ranging from one to 12).

Crime data for the years 2007-2008 were extracted from the Scottish Neighbourhood Statistics database [32]. This publicly available database contains data from various sources including the Scottish Census, police records and population surveys. Crime rates per 10,000 population were available at the datazone scale for selected crimes of violence, domestic housebreaking, vandalism, drug offences and minor assault. Total area-level crime rates were attributed to participants based on their residential datazone and were categorized as low, medium and high rates based on tertiles created using the entire sample distribution.

### Outcome variables

Participants were categorized as never, ex, or current smokers using the questions "Do you ever smoke tobacco now? (including pipe, cigars and roll ups)", and "If no, did you ever use to smoke any sort of tobacco?". Current smokers went on to report the number of cigarettes or cigars they smoked per day or per week for non-daily smokers, which we refer to as 'intensity'. Daily smoking intensity was analyzed as a continuous variable.

### Statistical analyses

Clustering of smoking across datazones was found not to be statistically significant in our sample. First, the intra-class coefficient from a null multilevel model including only smoking and datazones was non-significant. Second, results from logistic regression models and generalized estimating equation models with robust standard errors (often used to analyze correlated data [33]) were compared. Odds ratios and standard errors were nearly identical between the two sets of results, again suggesting non-significant clustering. We thus present results from logistic regression and general linear analyses carried with SPSS v.16.0. Logistic regression models were used to investigate the likelihood of being a current smoker as compared to a non-smoker (combining never and ex-smokers), given exposure to different levels of crime. The lowest crime category was used as a reference in all analyses. Linear regression models were used to estimate the adjusted mean number of cigarettes smoked daily across crime categories among current smokers.

Models were built in a step-wise fashion, separately for exposure to objective and perceived crime. Crime measures were entered first, followed by individual socio-demographic variables (sex, age cohort, SES, employment status). Perceived crime was entered last in the crime rate model, and vice versa. Analyses stratified by sex and age cohort were conducted to explore modification by these variables.

## Results

Table 1 presents descriptive statistics for participants according to their smoking status. Smoking prevalence was higher among the younger cohorts than among participants in the oldest cohort. There was a statistically significant gradient according to SES and neighbourhood crime rate, with higher proportions of current smokers belonging to lower social classes and residing in neighbourhoods characterized by higher crime rates. A significantly higher proportion of participants perceiving high crime in their neighbourhood were current smokers compared to those perceiving low crime in their area, among both males and females. Objective and perceived crime measures were not strongly correlated with Pearson correlation coefficients ranging between 0.20 among women and 0.25 among men (data not shown).

**Table 1:** Characteristics of study participants by smoking status

Individual characteristics	Total n	Current smokers (%)	**Mean number cigarettes/day**^**a **^**(SD)**
**Sex**			

Men	1,073	25.1	15.1 (8.6)

Women	1,345	23.5	14.3 (8.7)

Age cohort*			

30-50 years-old	829	28.6	13.3 (7.7)

50-70 years-old	953	25.9	16.6 (9.2)

> 70 years-old	636	15.9	13.0 (8.4)

Social class *			

I (professional)	248	11.3	13.4 (8.8)

II (intermediate)	803	16.3	11.3 (7.6)

IIINM (skilled non-manual)	350	21.4	14.6 (8.9)

IIIM (skilled manual)	634	33.0	16.0 (8.5)

IV (partly skilled manual)	293	34.5	15.7 (8.8)

V (unskilled manual)	90	46.7	16.9 (8.7)

**Employment status**			

Not in employment	1,032	24.6	15.3 (9.0)

In employment	1,386	23.9	14.2 (8.3)

**Neighbourhood crime**			

***Police-recorded crime***			

Crime rate (all respondents)*			

Low	805	15.8	13.8 (8.9)

Medium	800	25.3	13.8 (8.3)

High	813	31.5	15.8 (8.7)

Crime rate (men)*			

Low	371	17.0	14.9 (8.8)

Medium	332	26.5	13.4 (8.7)

High	370	31.9	16.4 (8.3)

Crime rate (women)*			

Low	434	14.7	12.7 (9.0)

Medium	468	24.4	14.1 (8.1)

High	443	31.2	15.3 (9.0)

***Resident-perceived crime***			

Perceived crime (all respondents)*			

Low	691	21.7	14.6 (8.2)

Medium	592	20.3	14.5 (9.0)

Medium-high	458	21.8	13.7 (8.1)

High	677	31.8	15.3 (9.1)

Perceived crime (men)*			

Low	308	21.4	14.8 (7.4)

Medium	257	24.1	14.9 (9.8)

Medium-high	198	22.2	14.0 (8.2)

High	310	31.3	15.8 (8.8)

Perceived crime (women)*			

Low	383	21.9	14.4 (8.7)

Medium	335	17.3	14.0 (8.0)

Medium-high	260	21.5	13.5 (8.1)

High	367	32.2	14.9 (9.3)

*n *sample size, *SD *standard deviation

^a^Among current smokers only

*Smokers and non-smokers differed significantly (*p-value *< 0.05)

Table 2 presents odds ratios and 95% confidence intervals for the association between neighbourhood crime measures and the likelihood of being a current smoker. Although interaction terms between crime measures and gender were statistically significant for perceived crime only (*p value *at the 0.05 significance level = 0.035, data not shown), we present results for all respondents and for men and women separately for both exposures, for reasons mentioned in the introduction. Adjusted Nagelkerke R^2 ^are presented as indicators of model goodness-of-fit. After adjusting for individual characteristics (model 2), residents living in areas characterized by high or medium crime rates had a statistically significant higher likelihood of being current smokers, compared to residents of low crime areas. The effect was slightly stronger among women than men for residents of high crime areas (O.R (95% C.I) of 2.09 (1.48-2.96) and 1.75 (1.21-2.54) respectively). Adjusting for perceived crime (model 3) only reduced the association by about 1% in men and 5% in all respondents and in women alone and results remained statistically significant. Participants perceiving the highest level of crime had a higher likelihood of being a smoker (O.R (95% C.I.) of 1.43 (1.11-1.84)), compared to those perceiving the lowest level of crime in their area, after accounting for individual characteristics (model 2). After stratifying by sex, the association remained statistically significant in women only (O.R (95% C.I.) of 1.51 (1.07-2.13)). Adjusting for police-recorded crime rate reduced the effect of perceived crime (model 3). Odds ratios still remained above 1.0, ranging between 1.12 and 1.36, but results were below the significance level.

**Table 2:** Association between crime measures and the likelihood of being a current smoker, OR^a ^(95% CI)

			Model 1^b^	Model 2^c^	Model 3^d^
	**Total n**	**n**_**cases**_	**OR (95% CI)**	**OR (95% CI)**	**OR (95% CI)**

***Police-recorded crime rate***					

**All respondents**					

Low	678	127	1.00	1.00	1.00

Medium	598	202	1.80 (1.39, 2.31)	1.68 (1.30, 2.18)	1.67 (1.29, 2.16)

High	557	256	2.45 (1.93, 3.12)	1.94 (1.51, 2.50)	1.84 (1.42, 2.39)

*Adjusted R*^*2*^			0.035	0.132	0.137

**Men**					

Low	308	63	1.00	1.00	1.00

Medium	244	88	1.76 (1.23, 2.54)	1.66 (1.13, 2.44)	1.64 (1.11, 2.43)

High	252	118	2.29 (1.62, 3.24)	1.75 (1.21, 2.54)	1.73 (1.18, 2.54)

*Adjusted R*^*2*^			0.032	0.165	0.167

**Women**					

Low	370	64	1.00	1.00	1.00

Medium	354	114	1.86 (1.33, 2.61)	1.68 (1.19, 2.39)	1.70 (1.20, 2.42)

High	305	138	2.62 (1.88, 3.65)	2.09 (1.48, 2.96)	1.95 (1.36, 2.78)

*Adjusted R*^*2*^			0.038	0.120	0.132

***Resident-perceived crime***					

**All respondents**					

Low	691	150	1.00	1.00	1.00

Medium	592	120	0.92 (0.70, 1.20)	0.94 (0.71, 1.24)	0.90 (0.68, 1.19)

Medium-high	458	100	1.01 (0.76, 1.34)	0.95 (0.71, 1.28)	0.88 (0.65, 1.18)

High	677	215	1.68 (1.32, 2.14)	1.43 (1.11, 1.84)	1.26 (0.97, 1.64)

*Adjusted R*^*2*^			0.018	0.123	0.137

**Men**					

Low	308	66	1.00	1.00	1.00

Medium	257	62	1.17 (0.79, 1.73)	1.22 (0.81, 1.85)	1.17 (0.77, 1.77)

Medium-high	198	44	1.05 (0.68, 1.61)	0.97 (0.62, 1.54)	0.89 (0.56, 1.41)

High	310	97	1.67 (1.16, 2.40)	1.30 (0.88, 1.93)	1.12 (0.75, 1.68)

*Adjusted R*^*2*^			0.013	0.156	0.167

**Women**					

Low	383	84	1.00	1.00	1.00

Medium	335	58	0.75 (0.51, 1.08)	0.75 (0.51, 1.11)	0.73 (0.49, 1.07)

Medium-high	260	56	0.98 (0.67, 1.43)	0.95 (0.64, 1.41)	0.88 (0.59, 1.31)

High	367	118	1.69 (1.22, 2.34)	1.51 (1.07, 2.13)	1.36 (0.96, 1.93)

*Adjusted R*^*2*^			0.025	0.117	0.132

*CI *confidence interval, *n *total sample size, *n*_*cases *_number of cases, *OR *odds ratio

^a^Never/ex-smokers serve as the reference category

^b^Model 1: Unadjusted (includes smoking and crime rate or perceived crime only)

^c^Model 2: Adjusted for sex, age cohort, SES and employment status; sex-specific models adjusted for these confounders excluding sex

^d^Model 3: Adjusted for sex, age cohort, SES, employment status and perceived neighbourhood crime (for objective crime model) and for objective crime (for perceived crime model); sex-specific models adjusted for these confounders excluding sex

Results of analyses stratified by age cohort are shown in Table 3. After adjusting for individual characteristics, participants from all three age cohorts living in a neighbourhood characterized by high police-recorded crime had a higher likelihood of being a current smoker, compared to those residing in low crime areas. Adjusting for perceived crime reduced the effect of crime on smoking among participants aged 30-50 years-old and rendered it non-significant. Perceived crime did not considerably affect the magnitude and significance level of odds ratios for participants aged between 50 and 70 years or those older than 70 years (O.R (95% C.I.) of 2.16 (1.45-3.24) and 2.29 (1.20-4.36) respectively). For measures of perceived crime, the only statistically significant results were found for the youngest cohort (fully adjusted O.R (95% C.I.) of 1.63 (1.04-2.55)).

**Table 3:** Association between crime measures and the likelihood of being a current smoker, by age cohort, OR^a ^(95% CI)

			Model 1^b^	Model 2^c^	Model 3^d^
	**Total n**	**n**_**cases**_	**OR (95% CI)**	**OR (95% CI)**	**OR (95% CI)**

***Police-recorded crime rate***					

**30-50 years-old**					

Low	224	59	1	1	1

Medium	179	76	1.61 (1.09, 2.39)	1.40 (0.93, 2.11)	1.35 (0.89, 2.05)

High	189	102	2.05 (1.41, 2.98)	1.58 (1.07, 2.35)	1.39 (0.92, 2.10)

*Adjusted R*^*2*^			0.025	0.138	0.149

**50-70 years-old**					

Low	276	52	1	1	1

Medium	234	84	1.91 (1.29, 2.81)	1.67 (1.11, 2.49)	1.66 (1.11, 2.48)

High	196	111	3.01 (2.06, 4.38)	2.24 (1.51, 3.33)	2.16 (1.45, 3.24)

*Adjusted R*^*2*^			0.053	0.144	0.146

**>70 years-old**					

Low	178	16	1	1	1

Medium	185	42	2.53 (1.37, 4.66)	2.31 (1.24, 4.32)	2.28 (1.22, 4.27)

High	172	43	2.78 (1.51, 5.12)	2.30 (1.22, 4.33)	2.29 (1.20, 4.36)

*Adjusted R*^*2*^			0.036	0.085	0.091

***Resident-perceived crime***					

**30-50 years-old**					

Low	220	52	1	1	1

Medium	210	51	1.04 (0.67, 1.61)	1.07 (0.68, 1.70)	1.04 (0.65, 1.65)

Medium-high	167	42	1.09 (0.68, 1.73)	1.05 (0.64, 1.70)	0.99 (0.60, 1.62)

High	232	92	2.12 (1.41, 3.19)	1.78 (1.16, 2.73)	1.63 (1.04, 2.55)

*Adjusted R*^*2*^			0.032	0.145	0.149

**50-70 years-old**					

Low	244	57	1	1	1

Medium	221	49	0.94 (0.61, 1.44)	0.98 (0.62, 1.54)	0.94 (0.59, 1.48)

Medium-high	179	43	1.04 (0.66, 1.63)	1.03 (0.64, 1.66)	0.95 (0.59, 1.53)

High	309	98	1.52 (1.04, 2.23)	1.37 (0.92, 2.04)	1.20 (0.80, 1.81)

*Adjusted R*^*2*^			0.012	0.126	0.146

**>70 years-old**					

Low	227	41	1	1	1

Medium	161	20	0.64 (0.36, 1.15)	0.71 (0.39, 1.28)	0.70 (0.39, 1.27)

Medium-high	112	15	0.70 (0.37, 1.33)	0.74 (0.38, 1.42)	0.70 (0.36, 1.36)

High	136	25	1.02 (0.59, 1.77)	1.12 (0.63, 1.97)	0.97 (0.54, 1.74)

*Adjusted R*^*2*^			0.009	0.069	0.091

*CI *confidence interval, *n *total sample size, *n*_*cases *_number of cases, *OR *odds ratio

^a^Never/ex-smokers serve as the reference category

^b^Model 1: Unadjusted (includes smoking and crime rate or perceived crime only)

^c^Model 2: Adjusted for sex, SES and employment status

^d^Model 3: Adjusted for sex, SES, employment status and perceived neighbourhood crime (for objective crime model) and for objective crime (for perceived crime model)

In terms of smoking intensity, Table 4 shows the F statistics and *p values *for significance of the difference between mean number of cigarettes smoked (unadjusted and adjusted) across exposure categories. Unadjusted models suggested that respondents living in areas with high crime rates or who perceived high crime in their neighbourhood smoked on average one to two and a half cigarettes per day more than those living in low crime areas. None of the associations between police-recorded or resident-perceived crime and smoking intensity were statistically significant at the 0.05 level, save for the unadjusted association between police-recorded crime rate and smoking intensity among all respondents. Analyses stratified by age cohort suggested that respondents aged 30-50 years-old and living in high police-recorded crime areas smoked on average more cigarettes than those residing in low or medium crime areas (statistically significant fully adjusted means of 12.24, 11.93 and 14.92 cigarettes/cigars per day for residents of low, medium and high crime areas respectively (data not shown)). Among the older two cohorts, none of the associations between police-recorded crime rate and smoking intensity reached statistical significance at the 0.05 level (data not shown). A high level of perceived crime was not associated with smoking intensity in models stratified by age cohort (data not shown).

**Table 4:** Mean number of cigarettes/cigars smoked per day^a^

		Model 1^b^		Model 2^c^		Model 3^d^	
***Police-recorded crime rate***	**n**_**cases**_	**Mean**	***p-value***^**e **^**(F)**	**Mean**	***p-value *(F)**	**Mean**	***p-value *(F)**

**All respondents**	570		0.02 (3.78)		0.16 (1.84)		0.18 (1.71)

Low	126	13.79		14.27		14.28	

Medium	196	13.79		13.93		13.93	

High	248	15.79		15.44		15.43	

**Men**	257		0.05 (3.01)		0.09 (2.40)		0.10 (2.31)

Low	63	14.89		15.21		15.26	

Medium	83	13.37		13.46		13.46	

High	111	16.41		16.16		16.12	

**Women**	303		0.14 (2.02)		0.62 (0.48)		0.66 (0.42)

Low	63	12.69		13.52		13.54	

Medium	113	14.10		14.21		14.22	

High	137	15.30		14.83		14.81	

***Resident-perceived crime***							

**All respondents**	570		0.75 (0.53)		0.75 (0.53)		0.59 (0.62)

Low	147	14.57		14.59		14.74	

Medium	117	14.49		14.66		14.70	

Medium-high	99	13.73		13.64		13.68	

High	207	15.27		15.20		15.06	

**Men**	257		0.44 (0.72)		0.64 (0.59)		0.58 (0.63)

Low	63	14.81		15.02		15.15	

Medium	60	14.93		14.86		14.90	

Medium-high	43	14.02		13.69		13.66	

High	91	15.79		15.85		15.74	

**Women**	303		0.34 (0.80)		0.31 (0.82)		0.21 (0.89)

Low	84	14.39		14.26		14.40	

Medium	57	14.02		14.35		14.38	

Medium-high	56	13.50		13.48		13.55	

High	116	14.86		14.81		14.66	

*n*_*cases *_number of cases

^a^Among current smokers only

^b^Model 1: Unadjusted (includes smoking and crime rate or perceived crime only)

^c^Model 2: Model adjusted for sex, age cohort, SES and employment status

^d^Model 3: Model adjusted for sex, age cohort, SES and employment status and perceived neighbourhood crime (for objective crime model) and for objective crime (for perceived crime model); sex-specific models adjusted for these confounders excluding sex

^e^*p-values *< 0.05 are suggestive of a statistically significant difference in mean number of cigarettes smoked daily by individuals across crime categories

## Discussion

In this study, residents living in areas characterized by high and medium police-recorded crime rates were more likely to be current smokers than residents of low crime areas. Similarly, individuals perceiving high crime in their neighbourhood were more likely to be smokers than those perceiving low crime levels. These associations remained statistically significant even after adjusting for individual characteristics. Odds ratios were faintly reduced after adjusting for either the objective or subjective crime indicator, suggesting that these might exert an independent effect on the likelihood of smoking. However, the perceived crime-smoking association was slightly more attenuated on adjustment for objective crime than the reverse, suggesting that part of the association between perceived crime and smoking is attributable to actual police-recorded crime rates in the neighbourhood. The association between both crime measures and smoking was more pronounced among women than men. A number of studies have previously found that area characteristics may be more strongly associated with women's smoking [11] or health in general [24]. This could be due to women being more aware of, or sensitive to, what happens in their neighbourhood [5]. Alternatively, women might be more exposed to their local area than men due to their spending more time there, on a daily basis [24]. In our study, high police-recorded crime rates were more strongly associated with being a current smoker among older respondents than among the younger cohort. A differential effect of neighbourhood exposure on the health of various age groups has been documented in other studies [34,35]. It could be attributable to older individuals being more exposed to actual crime rates in their residential neighbourhood given that they are often less mobile than the younger, working-age groups [26]. Older participants may also have had a longer lifetime exposure to their neighbourhood due to accumulating a longer residence time. Unfortunately, we did not have any information on the time participants actually spent in their neighbourhood on a daily or weekly basis, and we only had limited information relative to length of residence. Thus, we could not adjust for these variables in the models. We found the strongest effect for highest perceived crime to be among the younger participants. This could be due to younger groups' perceptions being more influenced by other factors, for example the media. Daily smoking intensity was slightly higher among residents of high crime areas or perceiving high crime in their neighbourhood in all groups except for the older age cohort. However, none of these results reached statistical significance save for the association between high police-recorded crime rate and smoking intensity among the youngest cohort.

This study is one of the few to have investigated the association between objective (police-recorded) and subjective (resident-perceived) crime measures and smoking. Our results concord with two studies which had reported an association between police-recorded crime and smoking [11,16]. The current findings of an association between high perceived crime and a higher likelihood of being a smoker add to the equivocal results found in the literature. Indeed, they concord with results from two studies having investigated perceived safety aggregated at the area level [17] or as an individual-based indicator [8] while two other reports had not found perceived safety to be associated to smoking [12,14].

To our knowledge, this study is the first to have examined the relative contribution of objective and subjective crime measures to the likelihood of smoking. Both indicators were associated with smoking status, however, when adjusting the crime rate-smoking association for perceived crime, or vice versa, estimates were slightly modified towards the null. This finding does not lend support to strong confounding, especially by the subjective crime measure of the association between objective crime and smoking. This is understandable in light of the low correlation coefficients between objective and perceived crime measures. This weak correlation mirrors that found in other studies [20,36] and suggests that these indicators may exert an independent influence on the risk of smoking. Crime rate and perceived crime are both chronic environmental stressors, defined as "insidious, with a slow and imperceptible onset and an open ended recurring character" [[37] p.2605], experienced at the neighbourhood level. They may be associated with stress and depression [37,38] and therefore influence smoking through stress-related mechanisms. However, objective crime rates may measure structural features not necessarily perceived by residents. Conversely, residents may not all be aware of, or influenced by, crime incidents in their local area defined with administrative boundaries [19,20]. As well, perceptions may be influenced by various factors other than actual crime activity such as residential tenure [25]. Thus, each crime indicator may measure neighbourhood features not encompassed by the other and capture neighbourhood characteristics which influence smoking through different mechanisms. For example, subjective measures of crime might be more proximal causes of stress and psychosocial disorder influencing health [19] and smoking, while objective crime rates may reflect other health-influencing neighbourhood features such as deprivation or lack of resources and amenities which might not be perceived negatively by residents [19] but which have also been found to be related to smoking.

This study presents a number of advantages and limitations. Important advantages include the large sample size, which allowed for exploring effect modification by sex and age cohort, and complete data for neighbourhood-level crime rates. Limitations are that smoking status was self-reported and determined using a question combining cigarettes and cigars, which could result in error in the measure of our outcome. However, prevalence data on cigarette versus cigar smoking indicate that cigar smoking is low in this population [39] so this may have a relatively minor influence on our results. In addition, former and never smokers were combined in the reference category for logistic models, and this may have led to exposure misclassification, diluting the effect of crime measures on smoking. Another limitation resides in a potential discrepancy between timing of exposure to crime and timing of its effect on smoking. Since no information was found concerning the lag time between exposure to crime and its effect on smoking, crime rates for the years 2007-2008 (i.e. when respondents were interviewed) were used. However, high correlations (>0.90) were found between 2007 and 2008 crime rates and those from earlier years (2001-2004) suggesting that crime rates are considerably stable over time. Therefore, exposure measured in 2007-2008 may be representative of an exposure having taken place earlier. An additional limitation concerns neighbourhood definitions. Although datazones are considerably small areas, they might not correspond to the neighbourhood respondents actually use and are exposed to, leading to exposure misclassification. In future research, more precise measures of exposure should be aimed for, for example by measuring exposure to crime within an area defined by participants, by taking into account the time they spend in their neighbourhood or by considering their exposure to other places where they spend time [40]. Finally, the data we used were cross-sectional, hampering the identification of a causal association between crime and smoking.

## Conclusion

In conclusion, high levels of police-recorded and resident-perceived crime measures were found to be associated with a greater likelihood of being a current smoker, over and above participants' individual characteristics. Attention should be paid to neighbourhood-level exposures, both objective and perceived by residents, to reduce smoking prevalence. Policy makers should thus focus on creating residential neighbourhood conditions which are less prone to crime, but which also make residents feel safe independently of actual crime rates in their area.

## Competing interests

The authors declare that they have no competing interests.

## Authors' contributions

MS conducted the statistical analysis and drafted the manuscript. Both authors participated in the interpretation of the data and contributed to subsequent drafts. Both authors read and approved the final manuscript.

## Pre-publication history

The pre-publication history for this paper can be accessed here:

http://www.biomedcentral.com/1471-2458/11/930/prepub
